# Availability of Tongue Diagnosis System for Assessing Tongue Coating Thickness in Patients with Functional Dyspepsia

**DOI:** 10.1155/2013/348272

**Published:** 2013-09-15

**Authors:** Juyeon Kim, Jiyoung Son, Seungwon Jang, Dong-Hyun Nam, Gajin Han, Inkwon Yeo, Seok-Jae Ko, Jae-Woo Park, Bongha Ryu, Jinsung Kim

**Affiliations:** ^1^Department of Clinical Korean Medicine, Graduate School, Kyung Hee University, Seoul 130-701, Republic of Korea; ^2^Department of Gastroenterology, College of Korean Medicine, Kyung Hee University, Seoul 130-702, Republic of Korea; ^3^Department of Biofunctional Medicine and Diagnosis, College of Korean Medicine, Sangji University, Wonju 220-702, Republic of Korea; ^4^Department of Statistics, Sookmyung Women's University, Seoul 140-742, Republic of Korea

## Abstract

Tongue diagnosis is an important procedure in traditional Korean medicine (TKM). In particular, tongue coating thickness (TCT) is deemed to show the progression of the disease. However, conventional tongue diagnosis has limitations because of various external factors. Therefore, it is necessary to investigate the availability of tongue diagnosis system (TDS) in the assessment of TCT. This study has been designed as a prospective clinical trial involving 60 patients with functional dyspepsia. Tongue images will be obtained by TDS twice with a 30 min interval. The system will measure the percentage of TCT and classify it as either no coating, thin coating, or thick coating according to the existing diagnostic criteria. After finishing the collection of 60 patients' tongue images, TCT on the images will be simultaneously evaluated by the conventional method to establish the gold standard for assessing TCT by 5 well-trained clinicians. The evaluation will be repeated by the same clinicians after 2 weeks, but the order of the images will be changed. This trial is expected to provide clinical evidence for the availability of TDS as a diagnostic tool and to contribute to the standardization of the diagnosis system used in TKM. This trial is registered with ClinicalTrials.gov NCT01864837.

## 1. Introduction

Tongue diagnosis is an important diagnostic procedure that is used in traditional Korean medicine (TKM) to examine the physiological function and pathological changes of the internal organs. It involves the examination of the body of the tongue (its color, shape, moisture, and movement) and the coating on the tongue (its color, thickness, distribution, and characteristics at the root) [1, 2]. The coating is believed to reflect the condition of the stomach as well as the nature and the site of the pathogenic factors. In particular, tongue-coating thickness (TCT) is considered to reflect the progression of a pathological condition [3]. A conventional method used in tongue diagnosis involves direct examination of a protruded tongue by a practitioner, so the result is not only affected by his or her subjectivity but also by environmental factors such as light [4]. Moreover, the available diagnostic criteria are insufficient and vague. 

To overcome the above limitations, a number of tongue diagnosis systems (TDSs) have been developed in recent years [5]. In general, TDS has a number of components including image capturing and storage, color correction, tongue segmentation, and image analysis. The processes of image capturing and storage are hardware resources, while those of color correction and tongue segmentation are image-preprocessing steps in tongue diagnosis. In the process of image analysis, the tongue is classified according to selected diagnostic parameters [5, 6]. In this study, tongues will be classified based on the measurement of TCT into those with no coating, thin coating, or thick coating according to the criteria developed by Kim et al. [7].

Functional dyspepsia (FD) is chronic or recurrent dyspepsia without evidence of organic disease [8]. The prevalence of FD is from 11.5% to 14.7% in the general population in Western countries [9], while in Republic of Korea, 25% of the population is affected as shown by a recent epidemiological survey [10]. We decided to include patients with FD in our study, because according to TKM, FD is one of the common stomach diseases that are well reflected by various features of tongue coating [11, 12]. 

For the objective and standardized tongue diagnosis system in TKM, it is necessary not only to develop an objective diagnostic tool but also to propose a new study protocol for clinical trial. We aimed to investigate the agreement between the results provided by TDS and those obtained by the conventional method in the assessment of TCT, which constitutes a part of tongue diagnosis in TKM, for evaluating on the availability of TDS in practice.

## 2. Materials and Methods

### 2.1. Study Setting and Participants

This study has been designed as a prospective clinical trial on the availability of TDS as a new diagnostic tool. Because there are no data available in the literature that would indicate the effective sample size to investigate the availability of TDS, we estimated the approximate sample size based on the number of outpatients with FD presenting at the Korean Medicine Hospital of the Kyung Hee University in Seoul, Korea. 

The study will include a total of 60 patients aged 20 years and older who meet the Rome III criteria for FD [8]. Patients complaining of dyspepsia within the previous 3 months and reporting the onset of symptoms at least 6 months prior to enrollment will be included in the study. They should have 1 or more of the following symptoms: bothersome postprandial fullness, early satiation, and epigastric pain and burning, without evidence of structural disease (on upper endoscopic examination) that could explain the symptoms. 

The exclusion criteria are as follows: pregnancy, mental disorders, severe systemic organ diseases such as cancer, infectious or other serious consumptive diseases, and geographic tongue. Patients unable to open their mouths or protrude their tongues; those unable to read, write, hear, or see; and those unable to sign a written informed consent form will also be excluded.

The study will be conducted in accordance with the standards of the International Committee on Harmonization of Good Clinical Practice and the revised version of the Declaration of Helsinki. The protocol of the trial has been approved by the institutional review board of the Korean Medicine Hospital of the Kyung Hee University (IRB no. KOMCIRB-2013-01). A written informed consent will be obtained from all patients before enrollment.

All patients will be required to visit the hospital twice during the study period. At the first visit, the written informed consent will be obtained, and patients will be screened for eligibility criteria. All eligible patients will be then instructed to avoid mouth rinsing as well as food and liquid intake for 4 hours before the examination with the TDS at the next visit. 

Tongue images will be obtained with the TDS under constant conditions at the second visit. Then, the percentage of tongue coating will be measured using a TDS analysis software, and TCT will be classified with the existing diagnostic criteria [7]. To assess the reproducibility of the TDS, the images will be taken again by the same method after 30 min. After two examinations of TDS, the weight of the tongue coating (mg) will be measured quantitatively to determine the correlation with the percentage of tongue coating. 

**Figure 1:**
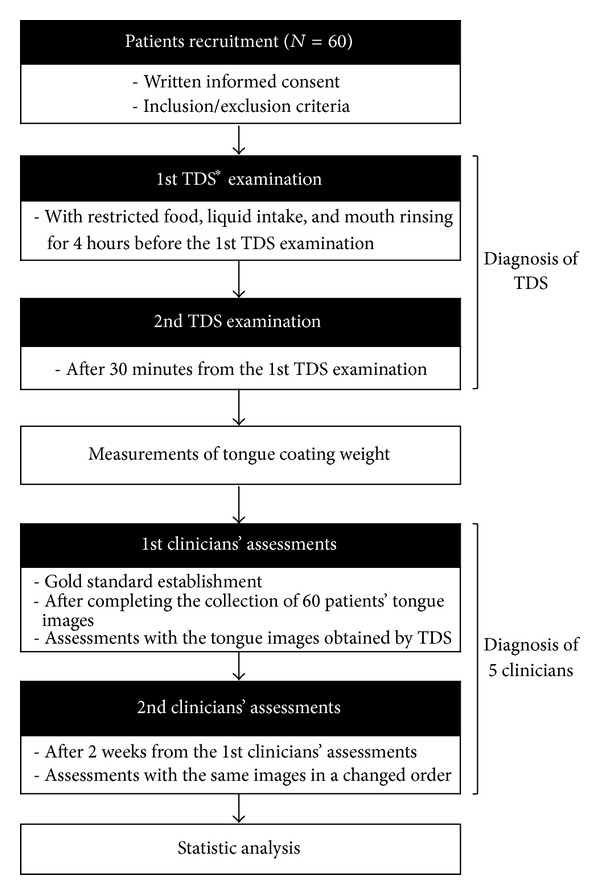
Flow chart of the trial. *TDS: tongue diagnosis system.

After we complete the collection of 60 patients' tongue images, the images will be simultaneously evaluated by 5 clinicians with 5 years or more of clinical experience to establish the gold standard for the assessment of TCT. The evaluation will be repeated after 2 weeks to analyze intra- and interrater reliability of the clinicians. Finally, we will evaluate the agreement between the results provided by TDS and those obtained by clinicians. The flow of the trial is shown in Figure 1.

### 2.2. Outcomes

#### 2.2.1. Primary Outcome

TDS will measure the percentage of tongue coating and classify TCT as either no coating, thin coating, or thick coating according to the existing diagnostic criteria [7]. At the same time, TCT shown on the images will be assessed by clinicians. We will then evaluate the agreement between the results of the TDS and the final diagnosis established by the clinicians.

#### 2.2.2. Secondary Outcome

To evaluate the performance of TDS, we will assess its reproducibility by comparing the 2 measurements of TCT in each patient and analyze the correlation between the percentage of coating measured with the TDS and the weight of coating obtained by tongue scraping [13]. Additionally, the intra- and interrater reliability will be analyzed to ensure the validity of the results obtained by 5 clinicians in the assessment of TCT.

### 2.3. Components of TDS

**Figure 2:**
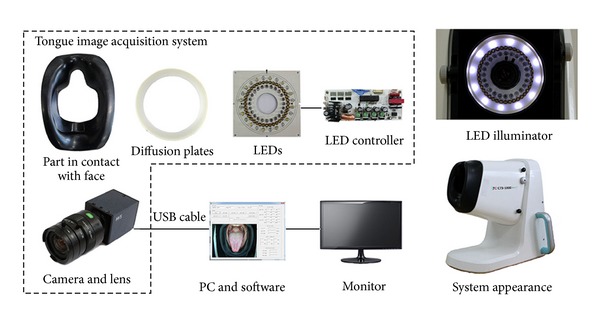
Components of a tongue diagnosis system (TDS).

The TDS consists of image acquisition system, LED illuminator, case, and analysis software. It is equipped with a vision camera (HVR-2130CPA, Hyvision System, Korea) and a mount lens (H2Z0414C-MP, Hyvision System, Korea). The camera can take color images of SXGA resolution (1280 × 1024 pixels) and automatically adjust white balance, exposure time, and gain settings. To evenly illuminate the dorsal surface of the tongue, 12 white LED lamps (*φ* 5 mm; first peak wavelength of 470 nm; second peak wavelength of 580 nm; color temperature of 6800 K) are arranged around the camera and thin double diffusion plates are placed in front of the lamps. The case is equipped with a part in contact with the face and a screw for adjusting the visual axis of the camera in order to take the tongue image at an appropriate position. The part in contact with the face ensures the proper location of the patient's nose and lower jaw and keeps the external light out of the TDS even if the facial appearance and degree of tongue protrusion change when the patient opens the jaws (Figure 2).

### 2.4. Image Analysis of TDS

**Figure 3:**
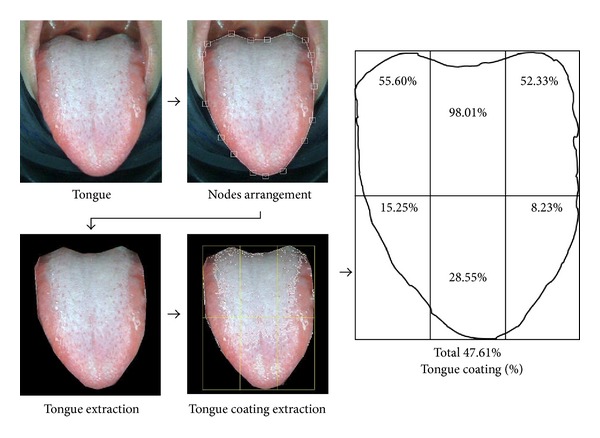
Schematic diagram for calculating tongue coating percentage by extracting tongue coating area.

An analysis software program of TDS was developed to analyze the percentage of tongue coating from the captured tongue images. To calculate the percentage of tongue coating, we first extract the tongue area from the captured tongue image. TDS generates 17 nodes on the tongue image on the monitor, and the operator moves the nodes on the boundary line of the tongue with a mouse. Then, the remaining area excluding the selected tongue area is removed. TDS converts the chromaticity coordinates of the extracted tongue image in the RGB color space to those in the CIE-Lab color space. The area of the tongue body is clearly more reddish than that of the coating. The positive a* value in the CIE-Lab color space is indicated by red/magenta, while the negative value by green. Therefore, tongue coating area is extracted from the entire tongue based on the difference in the a* value. The binarization threshold point of the a* value for the extraction was set through the tongue images acquired from the previous study [7] (Figure 3).

### 2.5. Measurement of TDS

Patients press their face against the interface of the TDS, open their mouth wide, and protrude their tongue for a second. An operator who is well trained in the use of TDS makes sure that the patient's tongue does not bend or lean to one side by watching the tongue image on the monitor and takes a picture of the tongue when ready. TDS allows to measure tongue coating percentage and classify TCT into 1 of 3 categories—no coating, thin coating, or thick coating—according to the diagnostic criteria developed by Kim et al. [7]. They developed differential criteria for TCT and specifically proposed a standard for the assessment of thin and thick coatings in tongue diagnosis. In their study, 24 clinicians who were well trained and highly reliable participated in the assessment of TCT from 50 tongue images obtained by TDS, which measured the percentages of tongue coating. From an analysis of a proportional odds model, they suggested a cutoff point of 29.06% to differentiate between no coating and thin coating and a cutoff of 63.51% to differentiate between thin coating and thick coating. It takes about 5 minutes from positioning his or her tongue to obtaining the diagnosis result by TDS for each patient.

### 2.6. Evaluation of System Performance

To assess the reproducibility of TDS, tongue images will be taken twice with a 30 min interval. Considering inevitable variations in length, angle, and shape of the protruding tongue in taking images, it will be necessary to have an appropriate interval between the two examinations to more accurately investigate the reproducibility of TDS. A 30 min interval will be able to prevent patients from intentionally protruding their tongues as they had done in the first examination. Then, we will compare TCT as shown on two different images for each patient.

After two examinations of TDS, we will measure the weight of the tongue coating with a method described by Yaegaki and Sanada [13] to analyze the correlation between the real quantity of tongue coating and the percentage of coating measured with TDS. The coating will be collected from each patient by the following procedure: first, cotton rolls will be put around the tongue to remove moisture; then, saliva on the dorsal surface of the tongue will be removed with a stream of air and pure pulp tissue paper; finally, the coating will be carefully collected with a tongue scraper from the terminal sulcus to the apex of the tongue; and the tongue dorsal surface will be cleaned with cotton pellets immersed in physiological saline. The weight of the coating will be then measured with a microbalance.

### 2.7. Conventional Method for Evaluating Tongue Coating Thickness

In the conventional method of tongue diagnosis, including the evaluation of tongue coating, a practitioner directly examines the tongue from the tip to the root. TCT is described as either no, thin, or thick coating. No coating literally means the absence of tongue coating; thin coating is diagnosed when the body of the tongue is barely visible; and thick coating when the body of the tongue cannot be seen at all [3].

### 2.8. Establishment of Gold Standard

To establish the gold standard for the assessment of TCT, 5 clinicians with 5 or more years of clinical experience and working at the Korean Medicine Hospital of the Kyung Hee University will be included in the trial to assess the images obtained by TDS. Before participation in the study, they will augment their knowledge about conventional methods of TCT assessment and be well trained in TCT differential criteria to obtain a reliable gold standard. The images will be sequentially presented to the clinicians using a picture-viewer software and an LCD monitor (DN-50PZ66, 50′′ HD, 1366 × 768 resolution, LG, Korea). Each image will be displayed for 10 seconds (10 minutes in total). Clinicians will be expected to label each image as either “no coating,” “thin coating,” or “thick coating.” Their assessment will be considered as a gold standard, and if there is a disagreement, we will choose the majority as the final diagnosis. All assessments will be performed individually in the same room and under the same conditions (in terms of brightness and distance between the clinician and the monitor). Clinicians will be blinded to the assessment of other clinicians and clinical data of the patients. The assessment will be repeated after 2 weeks and will involve the same clinicians, but the images will be presented in a changed order. The comparison of the 2 assessments will allow us to analyze the intra- and interrater reliability of the clinicians.

### 2.9. Statistical Analysis

The baseline characteristics of the patients will be presented as numbers and percentages for categorical variables and as mean ± standard deviation for continuous variables. Cohen's kappa (*κ*) coefficient will be calculated to analyze the agreement between the results of TDS measurement and the final diagnosis of the 5 clinicians as well as to assess the reproducibility of TDS. Fleiss' kappa (*κ*) coefficient will be calculated to evaluate the interrater reliability and Cohen's kappa (*κ*) coefficient to evaluate the intrarater reliability of TCT assessment by the clinicians. The results will be interpreted according to Landis and Koch (*κ* < 0.1 = poor; 0.1 ≤ *κ* ≤ 0.2 = slight; 0.2 < *κ* ≤ 0.4 = fair; 0.4 < *κ* ≤ 0.6 = moderate; 0.6 < *κ* ≤ 0.8 = substantial; 0.8 < *κ* ≤ 1.0 = almost perfect) [14]. The correlation between the weight and percentage of tongue coating will be analyzed using Pearson's correlation coefficient. All statistical analyses will be performed using PASW Statistics 18 (SPSS Inc., Chicago, IL, United States) and SAS software, version 9.1.3 (SAS Institute Inc., Cary, NC, United States). A *P* value less than 0.05 will be considered statistically significant.

## 3. Discussion

Tongue diagnosis is an important procedure in TKM, but conventional methods are limited by various external factors [4]. In an attempt to overcome these limitations, different types of TDSs have been recently developed [5]. The current study has been designed to investigate the availability of a newly developed TDS in the measurement of TCT by assessing the agreement between the results obtained by TDS and those obtained by well-trained clinicians as a gold standard. There have been several studies investigating diagnostic agreement and then emphasizing the importance of a training process based on the diagnostic criteria. Zhang et al. [15] showed in their clinical research that the training focused on a consensus of the traditional Chinese medicine (TCM) diagnostic criteria improved the agreement of TCM diagnosis in patients with rheumatoid arthritis. Son et al. [16] also investigated the importance of training in TCT assessment for the standardization of TKM tongue diagnosis. In their study, the agreement of TCT assessment among 15 clinicians after training was higher than that before training, and the agreement between TCT assessment and the value of TDS after training was also higher than that before training. Furthermore, the difference in agreement before and after training was significant (*P* < 0.05). Therefore, in the present study, to establish a reliable gold standard for TCT assessment and to improve diagnostic agreement, 5 clinicians will be sufficiently trained on the basis of TCT differential criteria by Kim et al. [7]. To our knowledge, this has been the first trial to compare TDS with a conventional method in the assessment of TCT for patients with FD. In addition, our study has been designed to investigate the reproducibility of TDS to provide a rationale for its wider use in clinical practice. Finally, the trial is expected to confirm the availability of TDS as an objective diagnostic tool. We believe that our results will contribute to the standardization of the diagnosis system used in TKM.

The study was designed in 2013. The first participant was enrolled in February, 2013. The recruitment of participants is ongoing. In addition, we have already finished the clinical trial registration (ClinicalTrials.gov NCT01864837).
